# Quick Estimate of Information Decomposition for Text Style Transfer

**DOI:** 10.3390/e25020322

**Published:** 2023-02-10

**Authors:** Viacheslav Shibaev, Eckehard Olbrich, Jürgen Jost, Ivan P. Yamshchikov

**Affiliations:** 1 Department of Intelligent Information Technologies, Ural Federal University, 620075 Ekaterinburg, Russia; 2 Max Planck Institute for Mathematics in the Sciences Leipzig, 04103 Leipzig, Germany; 3 CEMAPRE, University of Lisbon, 1649-004 Lisboa, Portugal

**Keywords:** text style transfer, natural language processing, information decomposition

## Abstract

A growing number of papers on style transfer for texts rely on information decomposition. The performance of the resulting systems is usually assessed empirically in terms of the output quality or requires laborious experiments. This paper suggests a straightforward information theoretical framework to assess the quality of information decomposition for latent representations in the context of style transfer. Experimenting with several state-of-the-art models, we demonstrate that such estimates could be used as a fast and straightforward health check for the models instead of more laborious empirical experiments.

## 1. Introduction

Natural language generation (NLG) is a challenging task. The discrete nature of textual information [1] leads to non-smooth disentangled representations and the absence of local information continuity [2] that make natural language generation even more complicated. One of the NLG tasks is style transfer for texts. This task is often addressed in the context of disentangled latent representations [1,3,4,5,6,7,8,9]. These works use an encoder–decoder architecture with one or multiple style discriminators to improve latent representations. An encoder takes a given sentence as an input and generates a style-independent content representation. The decoder then uses this content representation and a target style representation to generate a new sentence in the needed style (for a detailed review of modern text style transfer we address the reader to [10]).

There is a variety of benchmarks and methods used to compare the relative performance of the proposed architectures, see [11,12,13]. Yet, there is little rigorous work on how one could assess the quality of the resulting representations. For example, ref. [14] demonstrate that the quality of style and content decomposition depends on the particular architecture and models, with better information decomposition quality outperforming the state-of-the-art models in terms of BLEU (bilingual evaluation understudy) [15] between output and human-written reformulations. (BLEU’s output is always a number between 0 and 1. This value indicates how similar the candidate text is to the reference texts, with values closer to 1 representing more similar texts.) However, the experiments that illustrate this are very computationally intensive. This paper suggests assessing the quality of the obtained representations in a more effective, straightforward way and shows that the proposed theoretical estimates correspond to the empirical results. Since information decomposition within a latent representation might play a key role in minute text manipulation, ref. [1] hope that a computationally light model that assesses the quality of information decomposition in a given architecture could be instrumental for further research in natural language generation.

## 2. Related Work

The problem of text style transfer (TST) needs a more rigorous definition [16]. However, there are some attempts to quantify literary style, see [17]. Ref. [18] states that stylized texts could be generated if a system is trained on a dataset of stylistically similar texts. Ref. [19] show that the literary styles of the authors could be learned end-to-end.

Most recent contributions in the field address specific narrow aspects of style that could be empirically measured. Such stylistic attributes of text range from politeness [20], the *‘style of the time’* [17] and formality of speech [11] to author-specific attributes (see [21] or [22] on ‘shakespearization’), gender or political slant [23]. These attributes themselves are defined with varying degrees of rigor. Refs. [4,24] define a style as a set of arbitrary quantitatively measurable categorical or continuous parameters that could be automatically estimated with an external classifier. Further in this paper, we work with this empirical paradigm of literary style. It is widely used in modern text style transfer research since it allows natural extensions due to the compositionality of stylistic features. For example, ref. [12] provide a dataset for fine-grained stylistic changes as building blocks for more complex, high-level transfers, or [25] suggest treating style transfer as one-to-many mapping instead of one-to-one correspondence.

Many TST contributions either use an idea of an adversarial component to ensure that semantic representations contain no stylistic information [4] or combine it with some additional constrictions. For example, ref. [3] apply a GAN to align hidden representations of sentences from two corpora and use an adversarial loss to decompose information about the form of a sentence. Ref. [6] introduce adversarial–motivational training that includes a special motivational loss to encourage a better decomposition. Ref. [5] develop a structured content-preserving model that leverages linguistic information in the structured fine-grained supervision to preserve the style-independent content better. Ref. [7] show that the decomposition of style and content could be improved with an auxiliary multi-task for label prediction and adversarial objective for a bag-of-words prediction.

Recently, ref. [26] propose a new information-theory-motivated architecture for style transfer. They develop a method that leverages mutual information upper bound to measure dependence between style and content. Ref. [27] propose a method that can decompose speech into four components by introducing three information bottlenecks. The majority of the approaches mentioned above use some form of disentangled representation learning (DRL) [7], yet there are only a handful of methods to assess the relative quality of the obtained representations provided by different architectures. This paper addresses latent representation quality assessment in an information-theoretic framework called partial information decomposition. We propose a straightforward information-theory-based approach and demonstrate that the proposed estimates correspond to the empirical results but are significantly less computationally demanding.

In this paper, we experiment with a subtask of sentiment transfer. There is a discussion if the sentiment of a text could be regarded as its stylistic attribute, see [28]. However, numerous style transfer papers regard sentiment transfer as a viable task for the style transfer system. For example, refs. [29,30,31] estimate the quality of the style transfer with pre-trained binary sentiment classifiers.

## 3. Style Transfer

Consider the text style transfer that comprises an encoder–generator pair $M=\{f_{\theta_{\mathrm{enc}}},f_{\theta_{\mathrm{gen}}}\}$ parameterized by neural networks. The input to the model is a sentence $x=(w_{1},\cdots ,w_{T})$ where $w_{i}\in R^{d_{w}}$ and its style variable y∈{0,1}. The encoder maps x to a latent representation $z\in R^{d_{z}}$. The generator then takes z and *y* as inputs to generate a new sentence $\hat{x}$. Ideally, changing the value of *y* should generate $\hat{x}$ in a different style.

There are various methods for the evaluation of text style transfer models. For a detailed overview of modern semantic similarity measures and their applicability to the problem of style transfer, we address the reader to [32]. Instead of assessing the system’s overall performance, this paper focuses on the latent space that the style transfer model uses. Several TST papers state that precise text manipulation is enabled through effective information representation. However, there is no method that could compare the quality of latent spaces obtained by two different architectures. In this paper, we demonstrate that one can compare the rate of information decomposition achieved by a given model using measures from information theory.

## 4. Qualifying Latent Representations with Coinformation

Mutual information (MI) was originally proposed in Claude Shannon’s article “A Mathematical Theory of Communication” [33]. Given three jointly distributed random variables (X,Y,Z)∼P, the mutual information between *X* and *Y* and *Z* can be decomposed into information that *Y* has about *X* that is *unknown* to *Z* (we call this the *unique* information of *Y* w.r.t. *Z*) and information that *Y* has about *X* that is *known* to *Z* (we call this the *shared* or *redundant* information. Using the chain rule, the mutual information between *X* and (Y,Z) can be decomposed into four terms:

(1) $$\begin{array}{cc} \; & I(X;Y,Z)\\ \; & =\underset{\mathrm{unique}\;\mathrm{information}\;\mathrm{of}\;Y\;w.r.t.\;Z}{\underbrace{UI(X;Y\setminus Z)}}+\underset{\mathrm{shared}\;\mathrm{information}}{\underbrace{SI(X;Y,Z)}}\\ \; & +\underset{\mathrm{unique}\;\mathrm{information}\;\mathrm{of}\;Z\;w.r.t.\;Y}{\underbrace{UI(X;Z\setminus Y)}}+\underset{\mathrm{complementary}\;\mathrm{information}}{\underbrace{CI(X;Y,Z).}} \end{array}$$

This decomposition is part of a framework called *partial information decomposition (PID)* and was originally proposed by [34]. Ref. [35] made a now widely used proposal for concrete measures for the terms in Equation (1). The difference of the shared and synergistic information is equal to the *coinformation* [36,37], a symmetric measure of the correlation between three random variables:

(2) $$\begin{array}{c} CoI(X;Y;Z)=SI(X;Y,Z)-CI(X;Y,Z)\\ =I(X;Y)-I(X;Y|Z)\\ =I(Y;Z)-I(Y;Z|X)\\ =I(X;Z)-I(X;Z|Y) \end{array}$$

Coinformation is also widely used in neurosciences, with negative values interpreted as synergy and positive values as redundancy.

In the text style transfer (TST) setting, *X* represents the input text, *Y* its stylistic content and *Z* is the latent representation of the input that ideally should only capture the semantic content of *X*. In terms of the information decomposition in Equation (1) this would mean that when we decompose *X* there is unique information while the shared and the complementary information vanish. Since style *Y* is independent of latent representation *Z* given original input text *X*, the following statement holds

(3) $$\begin{array}{cc} CoI(X;Y;Z) & =I\left(Y;Z\right)-\underset{=0}{\underbrace{I(Y;Z|X)}}\\ =I(Y;Z). \end{array}$$

We propose to use I(Y;Z) as a proxy to measure the quality of the latent representation *Z*. Because Equation (3) implies that the shared information SI(X;Y,Z) is always greater than or equal to the complementary information CI(X;Y,Z), we can say that a successful text style transfer should transfer only the semantic aspect of X into Z and therefore has low I(Y;Z). In other words, these models should learn the latent representation *Z* in such a way that it keeps the redundant information SI(X;Y,Z) as low as possible.

## 5. Experiments

This section calculates the proposed I(Y;Z) for eight different style transfer architectures and shows how such a quantity can characterize the quality of the information decomposition in two given latent spaces.

### 5.1. Calculating Mutual Information

A framework for MI estimation between two continuous distributions was proposed [38]. This method is based on the estimator for differential entropy. In particular, one could apply the framework for the case where one of the distributions is continuous and another one is discrete [39] as follows

$$I\left(X,Y\right)=\psi \left(N\right)-\langle \psi (N_{x})\rangle +\psi \left(k\right)-\langle \psi (m)\rangle ,$$

where ψ(·) is the digamma function, 〈·〉 is the overall averaging of points from the dataset, *N* is the total number of points in the dataset, $N_{x}$ is the number of points for the given value *x* of discrete distribution, *k* is the number of nearest neighbors and *m* is the number of points which are closer than the *k*-th neighbor to the given point.

**Figure 1:**
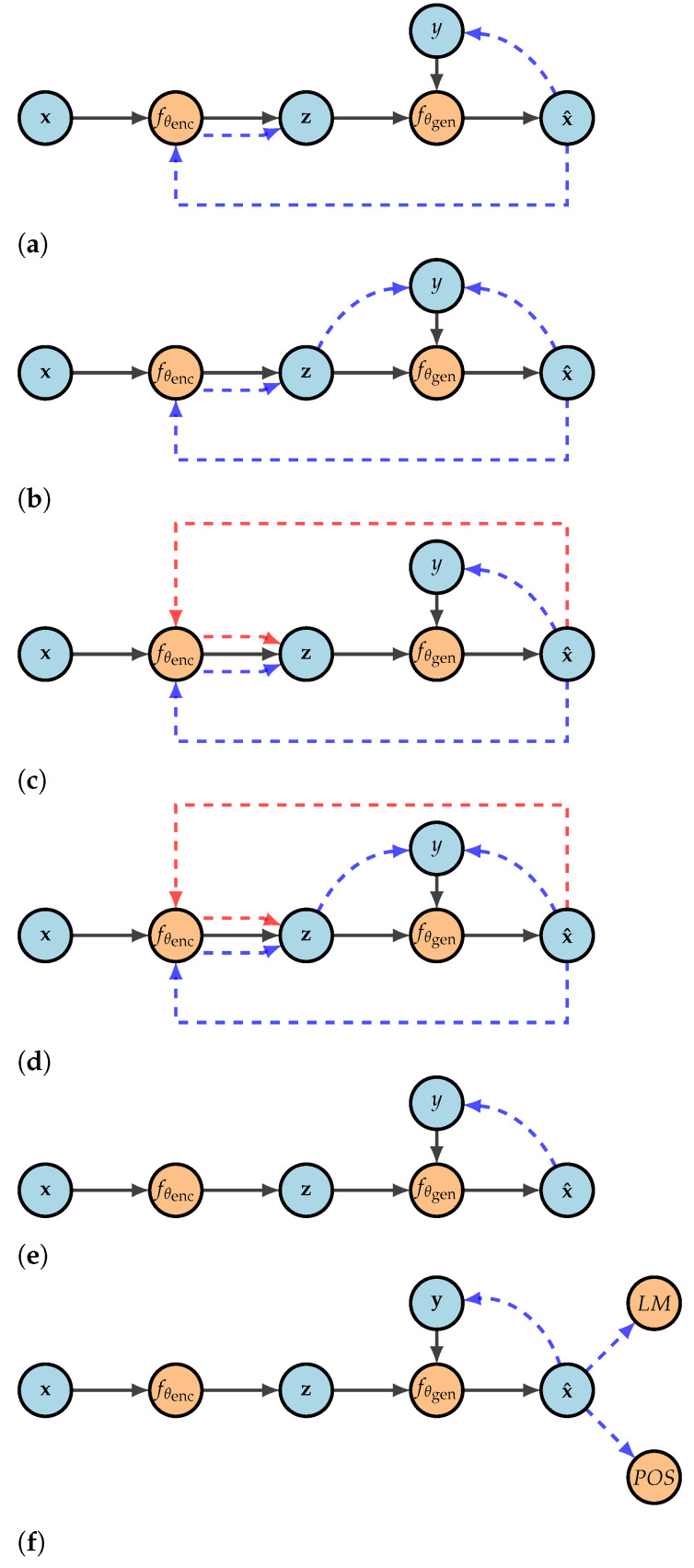
Text style transfer models evaluated in this work. Dashed lines indicate auxiliary training procedures that ensure z capturing the semantic content: the discriminators are denoted with blue color and the distance constraint is denoted with red. (**a**) **Baseline**: Variational autoencoder with discriminators [1]. (**b**) **ZDiscr**: Autoencoder with an additional discriminator [9]. (**c**) **SAE**: Shifted autoencoder [9]. (**d**) **SAEZDiscr**: Shifted autoencoder with an additional discriminator [9]. (**e**) **Shen et al. (2017)**: Aligned autoencoder [3]. (**f**) **Tian et al. (2018)**: Autoencoder with LM and POS losses [5].

We conduct our experiments on [5] human-rewritten Yelp! Reviews: the dataset contains 998 original and 998 reformulated Yelp! Reviews that are rewritten into either positive or negative sentiment. As shown in Figure 1, we experiment with six different text style transfer models, namely [1]’s autoencoder with discriminators that we further denote as (**Baseline**);autoencoder model with discriminators (**ZDiscr**), shifted autoencoder (**SAE**) and shifted autoencoder with discriminators (**SAEZDiscr**) introduced in [9]; Ref. [3]’s autoencoder for mapping texts written in different styles in the same latent space (**Shen**); Ref. [5]’s autoencoder with discriminator which trained with additional language model and part of speech losses (**TianFull**); a version of TianFull trained without additional language model loss (**TianWithoutLM**); and a version of TianFull trained without additional part of speech loss (**TianWithoutPOS**). In every figure, solid lines represent inputs and the dashed blue lines connect inputs compared by discriminators. In contrast, the dashed red line stands for the soft output of the architecture passed to the encoder to explicitly minimize the distance between latent representations of input and output.

Out of the architectures in question, only the one proposed by [3] does not use additional tools to estimate the quality of the output $\hat{x}$ to improve information decomposition in the encoder. Instead, it explicitly tries to minimize the distance between the aligned mapping of the sentences with different styles.

### 5.2. Exploring Latent Spaces

**Figure 2:**
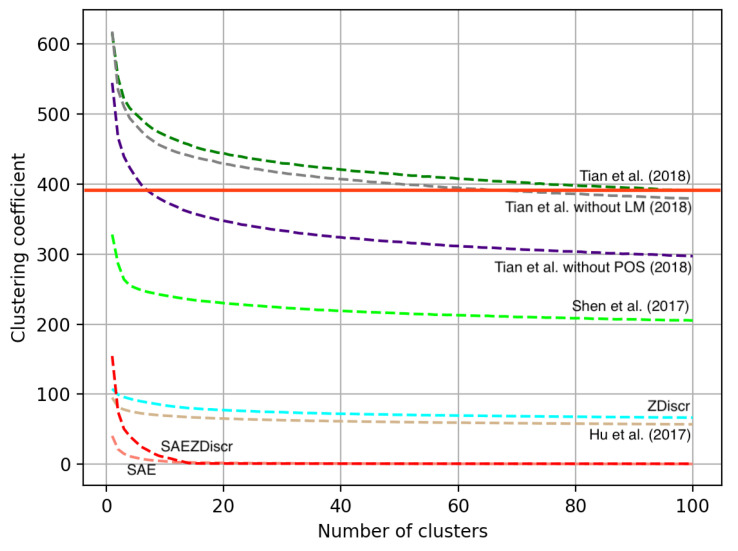
Clustering coefficient as a function of a number of clusters in a latent space obtained by architectures. Refs. [1,3,5].

Let us obtain some intuition on the underlying geometry of the obtained latent spaces. *k*-means clustering could be a straightforward and intuitive method to obtain such intuition. K-means, by default, uses a within-cluster sum of squares criterion. Since the estimate in Equation (4) uses nearest neighbors, it could be prone to noise depending on the structure of the resulting latent space. Figure 2 shows how the within-cluster sum of squares criterion changes for all architectures and their modifications as we choose different numbers of clusters. Figure 2 highlights vital differences between architectures. The lower clustering coefficient implies local dense clusters in a latent space, while the higher clustering coefficient implies that clusters are not dense. We see that some architectures such as SAE provide latent spaces with a smaller number of dense clusters, while others, like the model introduced in [5], obtain latent representations with no distinct structure, rather, latent representations form a cloud of points in the latent space.

**Figure 3:**
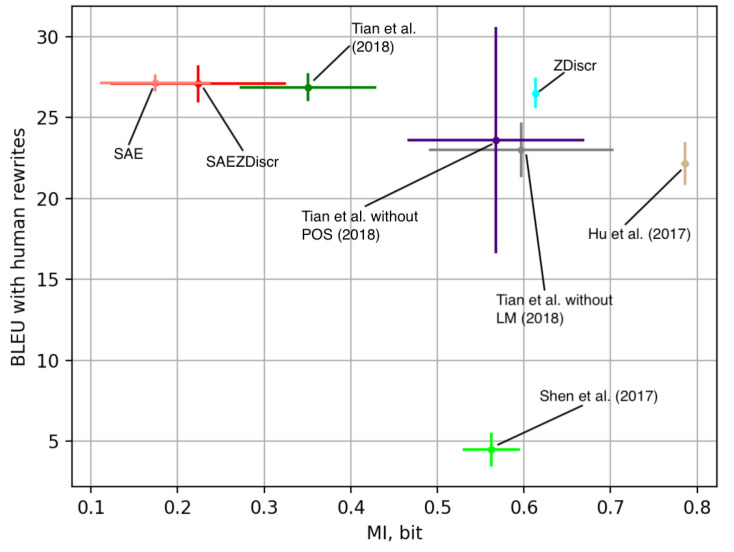
Estimated mutual information between target style and latent representation and BLEU between model output and human rewrites. Lower mutual information corresponds to higher BLEU and better performance. Refs. [1,3,5].

Having this basic intuition, let us now calculate a mutual information estimate according to Equation (4) setting the number of nearest neighbors to three as recommended in [39]. Figure 3 shows the obtained estimates of MI alongside BLEU scores between model outputs and human rewrites. Higher BLEU corresponds to better performance of the model. One sees that there is a general correspondence between the proposed MI estimate and the performance of the model. The nature of the models is different, and the results are prone to noise, yet there is a general tendency that models that achieve lower mutual information between stylistic variable and semantic representation tend to perform better in terms of BLEU between model outputs and actual human rewrites.

### 5.3. Correspondence with Empirical Results

In [14], the authors used a method proposed originally in [40] to see if better information decomposition corresponds to better style transfer performance. We reproduce and enhance these experiments here to see if our methodology aligns with these empirical results. The methodology originally proposed in [40] is as follows. To see if the encoder manages to decompose semantic and stylistic information, one can train a stand-alone artificial neural network that tries to predict the style of the input using its resulting latent representation. The lower accuracy of such classifiers corresponds to the higher quality of information decomposition. In [9], the authors show that the architectures used for style transfer are noisy. This means that to calculate the standard deviation for the results obtained with this method of information decomposition quality estimation, one has to retrain every architecture from scratch. Such experiments are computationally intensive and have to be tailored for every architecture.

**Figure 4:**
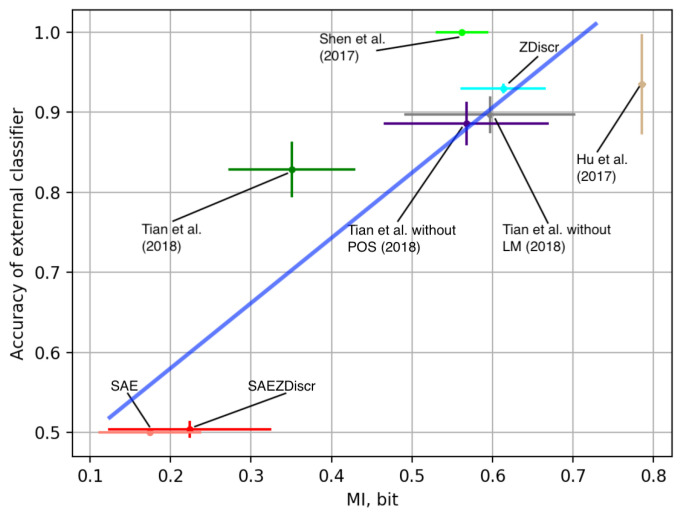
Accuracy of an external classifier and the proposed mutual information estimates, $R^{2}=0.78$. The proposed method for mutual information estimations obtains results similar to the previous empirical methods. Refs. [1,3,5].

Ref. [40] train a stand-alone artificial neural network that tries to predict the style of the input using its resulting latent representation. The authors suggest that the lower accuracy of such classifiers corresponds to a higher quality of information decomposition. Figure 4 shows the measurement of such external classifiers’ accuracy along with the estimates of MI proposed earlier in this paper. We run eight experiments with every architecture. Figure 3 and Figure 4 show standard deviations of the results along with the average numbers. One could see that the architectures with lower external classifier accuracy tend to deliver better performance in terms of BLEU with human-written rewrites. This means that measures for information decomposition quality could be useful for further advances in the research on minute language manipulation. However, this methodology demands the training of a stand-alone artificial neural network, and the results are prone to error due to inadequate choice of architecture. Naturally, if the architecture is not complex enough in comparison with the encoder, it could not provide adequate results, see [14]. However, comparing Figure 3 and Figure 4, one can see that our information-theory-inspired methodology gives results that are in line with the method proposed by [40] yet uses a fraction of computational resources and is not prone to the errors due to inadequate architecture design. One could use the proposed methodology as a simple and straightforward health check for the models that rely on information decomposition.

## 6. Discussion

Out of the eight models that we experiment with, there is one that stands out, namely, the method proposed in [3]. Figure 3 shows that despite the relatively low value of mutual information estimation, the model performs rather poorly in terms of the BLEU between human rewrites and the model’s output. Indeed, unlike other methods, [3] uses cross-alignment of the training data and does not provide any specific mechanism for estimation of the output quality as a part of the model. Instead, it tries to force distributional alignment over the latent space or sentence populations since the model is originally developed to work with nonparallel corpora rather than with style transfer on parallel texts. Thus, the model minimizes mutual information between *Y* and *Z* but does not explicitly maximize I(X,Y,Z). Under these structural assumptions, better information decomposition obtained does not guarantee better performance of the model, which we see in the experiments. This highlights the clear limitation of the proposed method: if the architecture does not explicitly maximize I(X,Y,Z), the decomposition quality assessment is not aligned with the performance of the model on the downstream task. Another explicit limitation of the proposed method corresponds to the applicability of the estimation methods proposed [38,39]: latent representation distribution has to be continuous while the distribution of the stylistic variable has to be discrete.

## 7. Conclusions

In this work, we have presented an alternative approach for evaluating the latent representation learned by TST models. If models learn the latent representations that capture only the semantic content of the inputs, the low value of mutual information between the latent representations and the target variable could measure the relative information decomposition quality obtained by various systems. Such methodology yields results in line with previous experiments yet is transparent and computationally efficient.
